# The Book World of Medicine and Science

**Published:** 1898-04-23

**Authors:** 


### The Book World of Medicine and Science.

Twentieth Century Practice of Medicine. Vol. X.—Diseases of the Nervous System. (London: 1897. Sampson Low, Marston, and Company. Pp. 859. Illustrated.)

The present volume of this valuable series is the first devoted to the nervous system, and is, with the exception of the articles by Dr. Féré, to be mentioned presently, exclusively the work of American authors. It opens with a general account of Diseases of the Brain, by Dr. Joseph Collins, of New York. This is carefully based on the latest histological and pathological researches, the main trend of which is clearly and succinctly stated. Dr. Collins's account of the neurons of which the brain is built up is most judicious, and compares favourably with any hitherto published in England. With regard to cerebral localisation, he is rather inclined to underrate the value of experiment, and falls into the curious error of believing the whole English school to be dominated by the teachings of Dr. Ferrier. Still, his views are in the main accurate, although not everywhere in accordance with the most recent additions to our knowledge. The section on thrombosis of the dural sinuses is worthy of particular attention, as this subject but rarely receives adequate notice. The article on Vascular Lesions of the Brain, by Dr. C. L. Dana, gives a good clinical account of these affections. Dr. B. Sachs writes with authority on Tumours of the Brain. Of these he considers that ophthalmological examination affords the most certain indication if it gives positive results; he admits, however, that cases occur fairly frequently in which optic neuritis is absent from beginning to end. His account of the localising symptoms of intracranial tumours is both lucid and concise, and bears evidence of much experience and careful thought. Diseases of the Meninges are described by Dr. Collins in a thorough and accurate manner; the account of epidemic cerebrospinal meningitis is surprisingly short, however, and throws but little new light on a disease which must be particularly interesting to Americans. The article on Hysteria, by Dr. Féré, is one of the longest in the volume. Much of it will probably be new to the English reader, particularly as regards the subject of stigmata, which is very fully treated; it may, indeed, be safely said that no more complete account of the subject has appeared in our language. The same author writes on Epilepsy and on the Spasmodic Neuroses, treating the former subject with the elegance and sound judgment for which he is noted, but erring rather in the direction of sketchiness in his handling of the latter. The volume concludes with an interesting article on the Disorders of Sleep, by Dr. Sanger Brown. Taken as a whole, it may fairly be said not only to sustain but to enhance the value of the stupendous work of which it forms part.

System of Diseases of the Eye. Vol. II. Edited by William F. Norris, A.M., M.D., and Charles A. Oliver, A.M., M.D., of Philadelphia, Pa., U.S.A. (London and Philadelphia, J. B. Lippincott and Co.: 1897. Pp. 556, with 13 full-page plates and 214 text illustrations. Price, by subscription, £1.)

The second volume of this magnificent work carries it halfway towards completion, and we can accord it no higher praise than to state that it is in every way a worthy successor of the first. The present volume treats of the examination of the eye, of school hygiene, of statistics of blindness, and of antisepsis. Mydriatics and Myotics are treated of by Dr. Snellen, jun., in a thoroughly scientific manner, the newer remedies being adequately considered as well as the old. The author dilates the pupil by preference by repeated instillation of ½ per cent. solution of atropine, together with boric acid. The article on the Ophthalmoscope and Ophthalmoscopy is by that well-known writer, Dr. G. M. Gould. It includes a very interesting historical account of the instrument, the author expressing his preference for the form designed by Morton. Skiascopy is treated of by Professor Jackson, whose monograph on the subject is a standard work, but the account here is somewhat sketchy and meagrely illustrated. Perimetry is fully discussed by Dr. Wilbrand, of Hamburg, and, indeed, occupies nearly a quarter of the book. The instrumental descriptions in this are unsatisfactory, Forster's old-fashioned apparatus being figured to the exclusion of Priestley Smith's, while no mention is made of the method of using the walls of a room as a perimeter recommended and adopted by Herring. Nor in the section devoted to affections of the field of vision in various diseases is there any reference to the most valuable studies of Dr. Buzzard in disseminated sclerosis and hysteria. Still, Dr. Wilbrand is on the whole very careful and accurate. The section which follows is on Colour-Blindness, and is from the pens of Professor William Thomson and Dr. Carl Weiland, the former of whom has long been admitted the leading authority on this subject in America. An eloquent plea is raised for State supervision of the question as affecting railway and naval officials, and is supported by experimental evidence of the risks run, and by records of accidents caused by colour-blindness in railway servants. Professor Risley's article on School Hygiene is perhaps the most important in the volume; it is divided into four sections, treating respectively of defects of vision in the schools, the necessity for an entrance examination, hygiene of the schoolroom, and school furniture; statistical tables, plans and diagrams of buildings and furniture, and illustrations of faulty positions are freely introduced, and we feel confident that this article will become quite a standard of reference in future. For the book as a whole we have, as already said, high praise; if the remaining volumes keep up to the standard so far reached its utility will be quite unsurpassed in its special branch.

Essentials of Obstetrics. By Charles Jewett, A.M., M.D., Sc.D., Professor of Obstetrics in the Long Island College Hospital; assisted by Harold F. Jewett, M.D. (Philadelphia and New York: Lea Brothers and Co. 1897. Pp. 385. Illustrated.)

This is by no means a cram book. Still, its authors do carry out the suggestion contained in its title, and limit their attention to what they consider essentials, leaving out of consideration much which adds greatly to the interest of any attempt to study the subject as a whole. But it is true enough, as the authors state, that the average medical student seldom has the necessary mental training to analyse the subject for himself, and that if he is well, even though dogmatically, grounded in the rudiments, a complete knowledge of the subject is facilitated. There is nothing special to note about the arrangement of the book. The anatomy is first dealt with, then the physiology of pregnancy, labour, and the puerperal state. Then come chapters on the pathology of these conditions, and a final chapter on obstetric surgery, in which the various obstetric operations are carefully described. Very full directions are given in regard to antisepsis and the use of antiseptic agents. Perhaps some of the methods inculcated are a little too elaborate for use in ordinary general practice; in fact, we are inclined to think that some portions of the methods advised, however desirable, are quite out of the question in many of the homes of the poor. It is somewhat of a defect that so little distinction is made between what is essential and what is desirable, for if a student is merely taught that all the details are to be gone through, he is apt to be completely at sea when he finds himself in a poor house without his sterilised coat, his sterilised water, his "surgically clean" rubber sheets, and all the rest. Nevertheless, it is on the whole a good book; the descriptions are clear, though short, and the teaching in regard to the employment of external examination and the avoidance of internal interference is sound.

